# Experimental Abdominal Sepsis: Sticking to an Awkward but Still Useful Translational Model

**DOI:** 10.1155/2019/8971036

**Published:** 2019-12-05

**Authors:** Federica Murando, Andrea Peloso, Lorenzo Cobianchi

**Affiliations:** ^1^General Surgery Department, Fondazione IRCCS Policlinico San Matteo, University of Pavia, Pavia, Italy; ^2^Hepatology and Transplantation Laboratory, Department of Surgery, Faculty of Medicine, University of Geneva, Geneva, Switzerland; ^3^Divisions of Abdominal and Transplantation Surgery, Department of Surgery, Geneva University Hospitals, Geneva, Switzerland; ^4^Department of Clinical, Surgical, Diagnostic and Paediatric Sciences, University of Pavia, Pavia, Italy

## Abstract

Animal models are widely used to replicate human intra-abdominal infections. Different methodologies have been described and proposed in the scientific literature, including injection and surgical models. The aim of this review is to recapitulate the advantages and disadvantages of each method to help choose the most appropriate model for individual experimental purposes.

## 1. Introduction

Sepsis and sepsis-related mortality remain severe problems in acute care units, despite the many advances over the years [1, 2]. Sepsis is progressively growing in everyday clinical practice in association with extremely high mortality, and severe complications can potentially occur, including trauma, burn, shock, and major surgery. Furthermore, it is also known to be one of the most important and frequent causes of death among critically ill patients [3].

In 2016, the consensus conference fine-tuned the latest definition of sepsis [4, 5] and defined it as a life-threatening organ dysfunction caused by a dysregulated host response to infection. The emphasis on life-threatening organ dysfunction underscores the need to recognise sepsis promptly and negate the need for the old term of “severe sepsis.” Another fundamental change concerns the method of recognition: the introduction of the SOFA score (Sequential “Sepsis-Related” Organ Failure Assessment) as a reference for the evaluation of organ dysfunction. The rating is based on six different items: respiratory, cardiovascular, hepatic, coagulation, renal, and neurological systems. Organ dysfunction can be identified as an acute change in the total SOFA score of ≥2 points resulting from the infection, which is associated with an in-hospital mortality risk of more than 10% [6].

Despite this last definition, sepsis remains a common, severe, and heterogeneous clinical entity that is difficult to define adequately. Animal models of sepsis have been examined to reproduce standardised pathophysiological changes in human sepsis. This method has helped to improve the understanding of the pathophysiology in humans and has allowed for the testing of new therapeutic agents. Many models have failed, while others have advantages and disadvantages (Table 1). Therefore, the topic is still under debate [7]. Currently, the search continues to find experimental models to capture the phenomena that occur in a human clinical setting as closely as possible.

## 2. Materials and Methods

This literature review examined the most relevant English language publications listed in PubMed in regard to the use of experimental animal models for abdominal sepsis that were published up to 2018. The search terms included the following: “animal models + sepsis,” “caecal ligation,” “caecal puncture,” “endotoxin,” “lipopolysaccharide,” “faecal pellet models,” “animal model + abdominal sepsis,” and “abdominal sepsis model.” Among the findings, a narrative review of the literature was performed with the aim of presenting and analysing the main animal experimental models of abdominal sepsis that are currently available.

## 3. Injection Models

### 3.1. Endotoxin/Lipopolysaccharide (LPS) Models

At the end of the 19^th^ century, endotoxins were first isolated by R. Pfeiffer, and in the early 1940s, Andre Boivin determined their activity [8]. Endotoxins are lipoprotein carbohydrate complexes that are present in the cell walls of Gram-negative bacteria. Borden et al. and Braude et al. later discovered the correlation between Gram-negative endotoxins and human septic shock through the lipopolysaccharide (LPS) model [9, 10]. This endotoxin-based model was the first attempted developed sepsis model. The model is based on the concept that sepsis could be caused not by the pathogen itself but could represent the final result of the host's response to bacterial products or endotoxins that have been intraperitoneally or intravenously injected. Endotoxins serve as a reasonable surrogate for bacteria, which makes a model simple to use and reproducible. However, therapeutic agents that were examined using this model proved to be unsuccessful when they were introduced into a clinical setting [11, 12].

To understand why this happens, it must be considered that the innate immune system of mice is activated after intraperitoneal or intravenous injections by the interaction among the bacterial product, LPS, and the cellular receptor CD14, which activate Toll-like receptors of the host's monocytes and macrophages. This process initiates both cell signalling and transcription of the inflammatory genes, leading to the production of inflammatory cytokines like TNF-*α*, IL-1, and IL-6. The way that LPS activates the cellular receptor is highly specific. Rodents, cats, and dogs are relatively resistant to endotoxins, while humans, rabbits, sheep, and nonhuman primates show an enhanced response.

When the dose of LPS is large enough, mice manifest biochemical and physiological changes that resemble certain fulminant human forms of Gram-negative bacterial infections. This acute endotoxaemia occurs in mice in the form of systemic arterial hypotension, impairs myocardial contractility, and increases circulating levels of TNF-*α* and IL-6. This response is the same as that of human endotoxaemia except for the different temporal kinetics and the magnitude of the physiological changes. In comparison to humans, mice are considerably less sensitive to the toxic or lethal effects of LPS, and therefore, they need higher doses of LPS to manifest the effects. Some factors that are present in the murine serum can neutralise the production of cytokines, which might explain the differences, but the nature of these factors is still unclear [13, 14]. Therefore, the discrepancy in the sensitivity to LPS between mice and humans suggests that the data obtained using mouse models of sepsis may be inapplicable to human disease.

**Figure 1:**
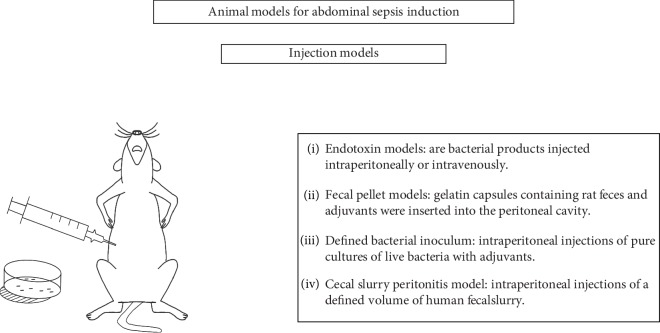
Injection models for abdominal sepsis induction.

Another fundamental difference between the LPS model and human sepsis is the cytokine release profile. In animals, a bolus injection of endotoxin usually causes an acute but transient increase in proinflammatory cytokines, such as TNF-*α*, IL-1, and IL-6. In human sepsis and the caecal ligation and puncture model (CLP), the host response is triggered by living bacteria, which provide much lower but long-lasting detectable levels of cytokines. Furthermore, from a hemodynamic perspective, endotoxaemia causes a hypodynamic state with reduced cardiac output and an increase in peripheral resistance, in contrast to sepsis, in which a hyperdynamic state occurs [15, 16]. Such findings suggest that the LPS model does not accurately reflect the sepsis of the CLP or human models (Figure 1).

### 3.2. Faecal Pellet Models

In the 1960s and 1970s, attention shifted from endotoxins to bacteria-induced sepsis. Faecal pellet models involve the intraperitoneal inoculation of gelatine capsules or fibrin clots containing faecal material and adjuvant substances like barium sulphate. With the aid of adjuvant substances, the dissolvable capsules prevent the rapid degradation of the inoculums and thus prolong the host's response. The first faecal pellet models were initially developed to study the differences between peritonitis and abdominal abscesses.

After the inoculation of faecal pellets, a two-step process occurs. In the initial step, acute sepsis develops, which has a mortality rate of about 40% after three days, and aerobic bacteria are predominant in the blood (mainly *E. coli* and enterococci). Among the surviving mice, the second step begins after five to seven days with the development of intra-abdominal abscesses, in which anaerobic bacteria such as *B. fragilis* and *F. varium* predominate [17, 18]. Based on this fact, faecal pellets offer a model of abdominal abscess following peritonitis rather than abdominal sepsis [19]. Furthermore, in various studies of this model, the mortality rate was found to be dependent on the type of faeces, which in turn depended on the feeding of the mice and the quantity of feed used [18]. Consequently, this model is uncontrollable and nonreproducible. Hence, the need persisted for the development of new sepsis models that use defined qualities and quantities of bacteria populations, which has prompted researchers to look for new models. Since then, established bacterial inoculum models have been developed.

### 3.3. Defined Bacterial Inoculum Models

Defined bacterial inoculum models involve the intraperitoneal inoculation of known quantities of bacteria mixed with faecal material or adjuvant substances [20–22]. The mortality of these models depends on the number of bacteria injected, the route of administration (intravenous, intratracheal, or intramuscular), and the fluids and antibiotics used. Although these models are controllable and reproducible, they have disadvantages [2, 11, 12, 16, 23, 24]. For example, if high doses of bacteria are injected, they do not colonise and cannot reproduce in the host because they are 157 lysed immediately by the complement system [23]. Thus, endotoxaemia is created rather than sepsis, which is why the bacterial inoculum must be injected with adjuvant substances that prevent its degradation [25, 26].

Coliform bacteria and anaerobes that are already sensed by faecal models have different roles. In 1976, Onderdonk et al. implanted different species of bacteria in mice either alone or in combination. They demonstrated that *E. coli* alone led to fulminant sepsis with early mortality, while mice that received a combination of both *E. coli* and *B. fragilis* developed more intra-abdominal abscesses [27]. This result was confirmed in subsequent experiments, such as those of Verweij et al. in 1991 [28].

### 3.4. Caecal Slurry Peritonitis Model

A team headed by Bauer developed a caecal slurry model of peritonitis in 2011 [29]. In an attempt to overcome the shortcomings of various models, they characterised a sepsis model using rats based on the intraperitoneal injection of a defined volume of faecal matter that has been obtained from three healthy nonvegetarian human donors. To standardise the protocol, they used aliquots from just one processed batch of frozen human stool to perform all the experiments, resulting in relatively low variability in terms of the infectious source.

Technically, the model is relatively easy to perform, which reduces the intrinsic variance of the surgical procedure. The following plan was carried out. First, 1.75 mL/kg body weight stool suspension was diluted (1 : 4) in saline and inoculated by a 21G cannula into the peritoneal cavity. Animals in the sham group received the same volume of saline. A survival analysis, clinical status evaluation, hemodynamic assessment, blood analysis, cytokine determination, rotation thromboelastometry (ROTEM), intravital microscopy, and histology of the liver were performed. At 40 h after the induction of sepsis, none of the animals (0/14) survived and showed a deteriorating disease condition beginning 2 hours after injection.

The hemodynamic analysis showed a decrease in the mean arterial pressure (MAP), arterial baseline excess, and PaCO_2_ with a concomitant rise in lactate levels. This biological profile clearly indicated a respiratory-compensated metabolic acidosis. The liver analysis revealed major hepatocellular dysfunction with a decrease of serum albumin levels in septic rats, followed by a severe degradation of haemostatic capacity. In parallel, intravital microscopy of the liver revealed excellent sinusoidal perfusion 5 h after the septic injury, but there was a 3-fold increase in the number of unperfused sinusoids, which was probably due to an interaction between leukocytes and the endothelium. As expected, the induction of peritonitis culminated in a rise in IL-6 and IL-10.

The proposed model demonstrated key features: it showed similar hemodynamic and physiological changes to those in human sepsis, it is reproducible, it is operator-independent, and it can be standardised. In a similar method, a caecal slurry model with faecal matter derived from sacrificed animals is suspended in liquid form and injected into the peritoneal cavity, and the results show hemodynamic and physiological changes that are comparable to human sepsis. This model has also been investigated by Lee et al., who analysed the effects of increasing volumes of peritoneal injections of caecal slurry and demonstrated dose-dependent mortality [30].

Polymicrobial sepsis is induced by the intraperitoneal injection of caecal slurry obtained from different donor rats. A total volume of increasing suspended caecal slurry (5.0, 7.5, 10, and 15 mL/kg) is administered through a 0.5 cm midline incision, and the survival rates that occur with various doses are compared. Sham-treated animals undergo the same volume injection of saline (without any caecal slurry). Polymicrobial-derived sepsis has been confirmed by recipient blood cultures, showing the presence of *Enterococcus faecalis* or *Enterococcus gallinarum*. All rats in the group receiving 0.5 mL/kg (*n* = 10) survived for 14 days, whereas all rats that received 15 mL/kg (*n* = 10) died within 24 h. Furthermore, 5 out of 30 rats (16.7%) receiving the 7.5 mL/kg died within 48 h with an overall mortality of around 40% within 14 days. These data confirm the dose-dependent mortality of this slurry sepsis rodent model.

## 4. Surgical Models

The first surgical models of sepsis were developed in the late 1960s and early 1970s. Bacterial contamination of the peritoneal cavity is the most frequent cause of septic peritonitis in humans. This could result from an intestinal leakage after surgery with the intraperitoneal extravasation of a large number of microorganisms present in the intestine (for this reason, such an infection is considered as polymicrobial in principle). Septic peritonitis is characterised by a major peritoneal infiltration of neutrophils and macrophages, which constitute the first line of defence for the elimination of bacteria. However, if they fail to limit the diffusion of peritoneal bacteria, they can reach the bloodstream and activate the cascading systemic immune response through the production of proinflammatory mediators, such as cytokines, which often leads to the onset of multiorgan dysfunction, septic shock, and death [31].

In early experiences with dogs and pigs, the model was based on the devascularisation of a segment of the intestine. The technique consisted of the ligation of the caecum below the ileocaecal valve while avoiding the interruption of intestinal continuity. The devascularised caecum undergoes necrosis with consequent transmural infection and the establishment of a septic scenario, but the results in terms of sepsis are not very clear [32–35]. The stability of the model was challenged and not very replicable due to the uncontrollable number of bacteria in the intestine, as well as the exact timing of bowel lesion.

In 1979, Keith et al. [36] demonstrated how caecal ligation alone did not lead to sepsis but to abdominal abscesses without sepsis in 71 mice. To resolve this problem, they proposed a new caecal ligation and puncture (CLP) model to improve upon previous models. CLP is based on the puncture of the caecum after its ligation below the ileocaecal valve to obtain continuous bacterial contamination of the peritoneal cavity. Their experiment showed that the effective development of sepsis is obtained with this model. Furthermore, this approach results in polymicrobial bacteraemia that is consistent with the same bacteria isolated at the site of infection and with the clinical signs of sepsis (fever, lack of appetite, and lethargy). This model shows the onset of peritonitis with polymicrobial flora, as well as devitalised/ischaemic tissue, thus mimicking clinical conditions such as appendicitis and diverticulitis [37].

All subsequent models have been inspired by CLP surgery, which still represents the gold standard of sepsis models. The mortality in this model and the increase in inflammatory mediators (IL-6 and TNF) depend on various factors, such as the diameter of the needle, number of perforations, length of the bound caecum, possible infusion, and antibiotic therapy [38]. The possibility of modulating the degree of severity of sepsis through these many variables was evaluated as an advantage of the model. However, there was a need to standardise the procedures to obtain consistent results. The versatility also represented a weakness of the model due to the lack of controllability because of the many variables [16]. It was also criticised for causing either fulminant sepsis or intra-abdominal abscesses among survivors, without always providing a clinical setting of generalised peritonitis [3, 39].

Since the first CLP model described in 1979, various versions have followed, which underscores how it still remains the gold standard despite 30 years having passed [40]. In 1997, Zantl et al. developed the colon ascendant stent peritonitis (CASP) model based on the insertion of a stent in the ascending colon. The idea behind this model arises from the principle of anastomotic dehiscences, which lead to peritonitis with subsequent septic shock and death. It was pointed out that as the stent diameter increased, mortality also increased. A 14-gauge (G) stent resulted in 100% mortality, while an 18 G stent caused 68% mortality, and a 22 G stent was fatal in 25% of treated animals [41, 42]. CASP leads to organ dysfunction as in septic patients and creates renal, pulmonary, and medullary damage with the production of IL-1 or IFN-gamma, as well as an independent TNF survival [42–46].

Maier et al. compared the CASP and CLP models and demonstrated that mortality is directly proportional to the diameter of the stent, but not to the number of punctures. Furthermore, in CASP, there is a high, constant increase in cytokines compared to CLP. Laparotomy performed at 24 h showed that faecal loss is maintained over time in CASP, while in CLP, there is a buffering of the focus with the formation of early ileal adhesions. The results once again revealed a pattern of intra-abdominal abscess formation with minor signs of systemic inflammation [47]. The disadvantage of CASP, however, is that bacterial contamination is continuous and not controllable.

To investigate the pathophysiological mechanisms of sepsis further, Zantl et al. later improved their model by creating surgical intervention after the CASP surgery (CASPI) model to determine whether surgical removal of the septic focus would prevent a lethal outcome after the induction of peritonitis and, if so, for how long. In this model, a relaparotomy is performed with stent removal and suture of the defect on the caecum at standardised times (after three, five, and nine hours), which mimics what is actually done in clinical practice for peritonitis. Stent removal after three hours resulted in 100% survival, while CASPI after nine hours showed 100% lethality. Surgery after five hours led to average mortality rates. These results clearly showed that critical pathophysiological events occur between three and nine hours after the induction of sepsis and develop independently of the continuous bacterial contamination of the peritoneal cord [42].

In 2009, Scheiermann et al. created the caecal ligation and incision (CLI) model to reflect severe sepsis characterised by acute onset and high mortality. In this model, caecum ligation is performed, and a 1.5 cm incision is made to establish a continuous leakage of faeces, which is directly inspired by the CLP model. They also wanted to obtain acute and severe sepsis as in models with endotoxins while overcoming their artificiality [48].

**Figure 2:**
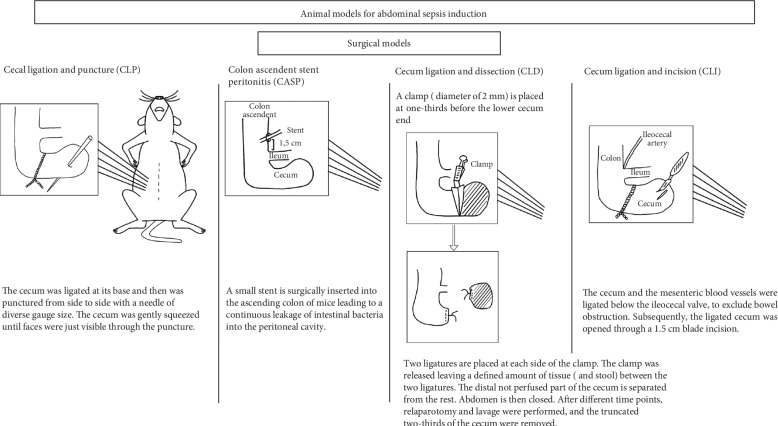
Surgical models for abdominal sepsis induction.

In 2013, the caecum ligation and dissection (CLD) model was created based on a 2 mm clamping of the last third of the caecum and its section after binding. A standardised quantity of faeces is released in the abdomen (2 mm^2^). The stump is abandoned in the abdomen, and then a relaparotomy is performed with the extraction of the piece of caecum, which is necrotic by this point, followed by antibiotic therapy. This 246 model is aimed at simulating what happens in clinical reality as closely as possible. According to the guidelines, sepsis due to intestinal perforation or dehiscence of anastomosis must be treated surgically with concomitant antibiotic therapy. This model presents great novelty in having a standardised quantity of loose stool in the abdomen and a free necrotic stump that cannot be encapsulated [39], as shown in Figure 2.

## 5. Discussion

The use of animals for scientific purposes is both a longstanding practice in biological research and medicine [49] and an ordinary matter of debate in our societies. The remarkable anatomical and physiological similarities between humans and animals, particularly mammals, have prompted researchers to investigate a broad range of biological mechanisms and assess novel therapies in animal models before applying their discoveries to humans. However, not all results obtained with animals can be directly translated to humans.

Naturally, the best approach would be to use animals that are very similar to humans, such as primates, but there are many ethical and economic limitations in this regard. Therefore, various types of animals have been used over the years. Dogs have mainly been used for surgical models, while rabbits have been used for some models of peritonitis, despite having some disadvantages. To date, mice remain the best animal model since they are available in large numbers with the same sex, age, and genetic heritage [35]. However, many limitations of animal models remain. Although rodents have many characteristics that are common to mammalian biology, there are many significant physiological and pharmacological differences with respect to humans. For instance, rodents (as well as cats and dogs) are moderately resistant to endotoxin, while humans, primates, rabbits, and sheep are highly sensitive.

In general, animals are carefully chosen to have a genetic background, age, weight, and nutritional status that are as similar as possible, which means that they do not reflect the heterogeneity among humans. Additionally, the pathophysiological progression of animal sepsis is very different from that of humans. Compared to humans, animals have a rapid onset of hypodynamic circulatory failure, with mortality in just a few hours. Furthermore, the animals that are usually selected are always young and healthy and do not reflect the actual age of incidence of sepsis among humans [50, 51]. In laboratories, there is no standardisation regarding the use of antibiotics, the infusion of liquids, or the type of resuscitation, which clearly leads to an increase in survival and variation in the results from one experiment to another [52].

In this field of research, whenever a new compound is studied in vitro and turns out to be effective, the next step is an experimental study in vivo. In this crucial translational passage, there is a need for experimental models of sepsis that are effective, repeatable, and as similar as possible to the clinical setting [7]. Hundreds of biological interventions have proven to be effective in animal models, but translation to humans has failed [2]. This problem is likely related to several factors, but the tremendous biological complexity of sepsis in particular makes it difficult to create ideal experimental models that are repeatable and similar to clinical situations in terms of pathophysiology. Animals used in experimental models are healthy when the sepsis starts, and compared to humans, many conditions are different and profoundly affect the translation, such as antibiotics therapies and intensive care support (mechanical ventilation, sedation, and analgesia).

## 6. Conclusions

The principal reason for investigating different animal models of abdominal sepsis is ultimately to develop retest therapies and protocols to treat sepsis in a clinical setting. Therefore, clinical research must account for every possible caveat of the model being used. Animal models are essential for scientific advancement in many areas of human health, but if they are not well characterised and understood, erroneous conclusions may be drawn, which hinders scientific progress and results in a waste of animal life.

A well-designed animal model requires a thorough understanding of the similarities and differences in the physiology of humans and animals and must incorporate knowledge into the goals of the study. Researchers should remember that all uses of experimental animals must take into account the animal welfare and the three “Rs” (refinement, reduction, and replacement). To that end, the information presented in this study provides a systematic basis for the development of animal models for abdominal sepsis research.

## Figures and Tables

**Table 1:** 

Models	Advantages	Disadvantages	References
Endotoxin model	Simple to perform, reproducible and controllable, high cost	Produces endotoxic shock and not sepsis; different from human sepsis in terms of kinetics and amplitude of cytokine production	[8–16]
Fecal pellet model	Simple to perform, high cost	Uncontrollable, nonreproducible: depending on the type of faeces (depending on feeding of the mice) and on the quantity used; undefined qualities and quantities of bacteria populations; fulminant sepsis vs. survival with intra-abdominal abscesses	[17–19]
Defined bacterial inoculum	Simple to perform, reproducible and controllable with type and quantity of bacteria population	Needs adjuvant substances; lack of pathophysiological mechanisms of the intestinal damage, fulminant sepsis vs. survival with intra-abdominal abscesses	[2, 11, 12, 16, 20–28]
Caecal slurry peritonitis	Simple to perform, reproducible, standardisable, similar to human sepsis	No disadvantages	[29, 30]
CL (caecal ligation)	Simple to perform	Uncontrollable: depends on the amount of bacteria in the intestine and the timing of bowel rapture	[32–36]
CLP (caecal ligation and puncture)	Better clinical relevance; lower cost; adjustable with the diameter of the needle, the number of perforations and the length of the bound cecum	Uncontrollable because of too many variables; fulminant sepsis vs. survival with intra-abdominal abscesses; no clinical setting of generalized peritonitis	[3, 16, 37–39]
CASP (colon ascendant stent peritonitis)	Adjustable with the diameter of the stent; generalized peritonitis and no abscesses obtained	Uncontrollable: bacterial contamination is continuous	[40, 47]
CLI (caecal ligation and incision)	Model for severe acute sepsis, overcomes the artificiality of the endotoxin model	Acute onset and high mortality	[48]
CLD (cecum ligation and dissection)	Standardisable: quantity of faeces is 2 mm^2^; surgical treatment: more close to clinic, long survivor with septicemia	No disadvantages	[39]
